# 1714. Incidence of Sexually Transmitted Infections in Youth with HIV Pre-COVID and COVID Era

**DOI:** 10.1093/ofid/ofad500.1547

**Published:** 2023-11-27

**Authors:** Firdous Khan, Leah Loerinc, Amy Scheel, Scott Gillespie, Andres Camacho

**Affiliations:** Medical College of Georgia, Athens, Georgia; Children's Hospital of Philadelphia, Philadelphia, Pennsylvania; Children's Hospital of Philadelphia, Philadelphia, Pennsylvania; Emory University School of Medicine, Department of Pediatrics, Atlanta, GA; Emory University School of Medicine, Atlanta, Georgia

## Abstract

**Background:**

Adolescents and young adults (AYAs) living with HIV have high rates of co-sexually transmitted infections (STIs). During the COVID pandemic, STI prevention strategies including access to testing and treatment facilities, availability of public health practitioners and healthcare workers, and condom availability may have decreased. The aim of this study was to determine if differences in STI incidence for first infection and re-infection existed between the pre-COVID and COVID eras in a cohort of AYAs living with HIV in Atlanta, GA.

**Methods:**

Retrospective chart review was conducted for all patients aged 13-24 at the Grady Ponce and Family Youth Clinic in Atlanta, GA. Two eras were identified: a pre-COVID era from 1/1/2009 – 12/31/2019 and a COVID era from 1/1/2020 – 6/30/2021. Patients could be followed in both eras, where follow-up for first and recurrent STI incidences were reinitiated at the start of the COVID era. STIs recorded included gonorrhea, chlamydia, human papillomavirus, syphilis, trichomonas, herpes simplex virus, lymphogranuloma venereum, hepatitis C, bacterial vaginosis, and chancroid. First and recurrent incidence rates for any STIs were reported.

**Results:**

Our sample included 766 sexually active AYAs with HIV. The mean age of first observation was 18.91 (±2.86) years. 72.3% of our patients were male and 90.7% were black. 721 patients were included in the pre-COVID era and 583 (80.9%) had at least one STI. 337 patients were included in the COVID era, and 158 had at least one STI (46.9%). The overall first STI incidence rate increased from 42.47 to 58.67 per 100 person-years from the pre-COVID to the COVID era (p< 0.001). Gonorrhea, trichomonas and syphilis had significantly higher rates during the COVID era (Table 1). The recurrent STI incidence rate for any STI also significantly increased from 121.50 to 169.85 per 100 person-years (p< 0.001) (Table 2).

**Figure:**
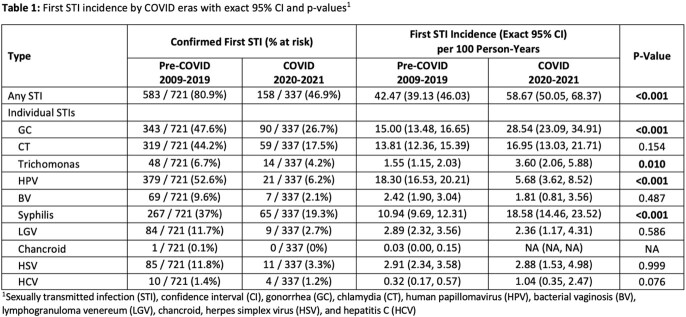

**Figure:**
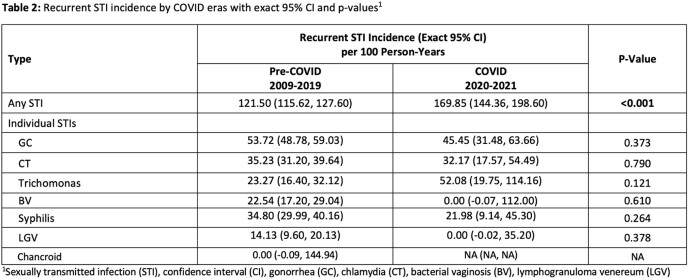

**Conclusion:**

Our study demonstrated significantly higher incidence rates of first and recurrent STIs in AYAs living with HIV in the COVID era compared to the pre-COVID era. Reallocation of resources during pandemics should emphasize continuation of existing STI prevention programs to avoid secondary clinical and economic adverse effects of increased infections.

**Disclosures:**

**All Authors**: No reported disclosures

